# Effects of sulfadiazine and Cu on soil potential nitrification and ammonia-oxidizing archaea and bacteria communities across different soils

**DOI:** 10.3389/fmicb.2023.1153199

**Published:** 2023-05-15

**Authors:** Guoqin Hou, Zafran Gul Wazir, Jing Liu, Guizhen Wang, Fangxu Rong, Yuzhi Xu, Mingyue Li, Kai Liu, Aijv Liu, Hongliang Liu, Fayuan Wang

**Affiliations:** ^1^School of Agricultural Engineering and Food Science, Shandong University of Technology, Zibo, China; ^2^School of Resources and Environmental Engineering, Shandong University of Technology, Zibo, China; ^3^School of Life Sciences and Medicine, Shandong University of Technology, Zibo, Shandong, China; ^4^School of Environment and Safety Engineering, Qingdao University of Science and Technology, Qingdao, China

**Keywords:** sulfadiazine, copper, toxicity, soil potential nitrification, AOA, AOB, soil type

## Abstract

**Introduction:**

Sulfadiazine (SDZ) and copper (Cu) are frequently detected in agricultural soils, but little is known on their single or combined impact on ammonia oxidizing microbial community and function across different soils.

**Methods:**

In this study, a microcosm was conducted to distinguish the microbial ecotoxicity of SDZ and Cu across different soils by analyzing soil potential nitrification rate (PNR) and the *amo*A gene sequences.

**Results:**

The results showed that the single spiking of SDZ caused a consistent decrease of soil PNR among three tested soils, but no consistent synergistic inhibition of SDZ and Cu was observed across these soils. Moreover, across three tested soils, the distinct responses to the single or joint exposure of SDZ and Cu were found in *amo*A gene abundance, and diversity as well as the identified genus taxa of ammonia-oxidizing archaea (AOA) and bacteria (AOB). Meanwhile, only the specific genus taxa of AOA or AOB consistently corresponded to the variation of soil PNR across different treated soils. The further principal component analysis (PCA) exhibited that the variable influence of SDZ and Cu on ammonia oxidizing microbial community and function was greatly dependent on soil type.

**Discussion:**

Therefore, in addition to ecological functionality and the specific prokaryotic taxa, soil microbial ecotoxicity of SDZ and Cu also was dependent on edaphic factors derived from soil types. This study proposes an integrative assessment of soil properties and multiple microbial targets to soil contamination management.

**Graphical Abstract:**
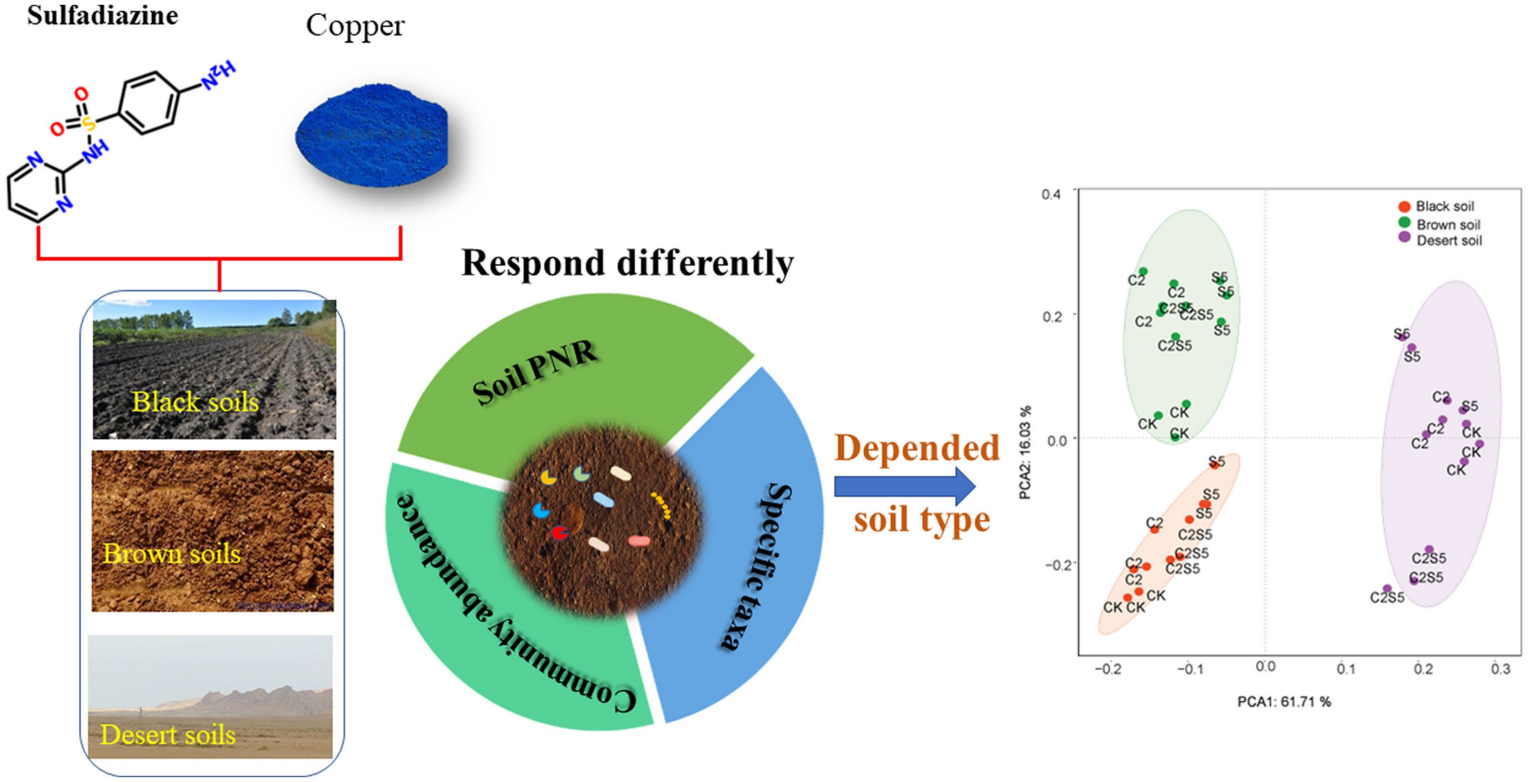


## Highlights

- PNR and *amoA* genes responded differently in soils exposed to SDZ and Cu.
- The impacts of SDZ and Cu on AOA and AOB communities depended on soil types.
- The specific phylotype corresponded to the PNR variation under the stress of SDZ and Cu.

## 1. Introduction

Recently, increasing attention is paid to soil contamination by veterinary antibiotics and heavy metals (Guo et al., 2018), especially their combined toxic effects on soil microbial ecology (Xu et al., 2016; Grenni et al., 2018; Wei et al., 2018; Wang L. et al., 2019). Moreover, a few investigations found that combined treatments of antibiotics and heavy metals produced higher toxicity on soil microbial ecology than the single one (Xu et al., 2016; Wang L. et al., 2019), which were strongly dependent on the added ratio, exposure time (Yang et al., 2020), and the specific biological indicator (Liu et al., 2015). It was reported that the co-existence of heavy metals and antibiotics could change soil ecological function by shifting microbial community structure (Cycoń et al., 2019). In light of the pivotal roles in the nitrogen cycling of ecosystems (Falkowski et al., 2008; Ke et al., 2013; Wang et al., 2015), ammonia-oxidizing microorganisms and the compounding function were widely chosen as biomarkers of soil environmental contamination (Katipoglu-Yazan et al., 2016; Zhang et al., 2017; Wei et al., 2018). It was found that combined exposure to antibiotics and heavy metals caused stronger toxicity than the single one on ammonia-oxidizers’ abundance and nitrification activity (Liao et al., 2019a,b; Wang L. et al., 2019). The abundant data also indicated that the entered toxicant could alter soil microbial activity (Gil-Sotres et al., 2005; Hammesfahr et al., 2011; Cycoń et al., 2016) and then shift their community structure (Wang L. et al., 2019). However, it was reported that the functional redundancy and the specific phylotypes modulate the contribution of soil microbial diversity and composition to multifunctionality (Li et al., 2021). Thus, it still could be debated whether or how soil contaminants caused real toxic effects on soil nitrification. This calls for further investigation to differentiate the responses of soil nitrification function and ammonia oxidizing microorganisms under contaminated stress; more importantly, the ecological mechanisms in mediating these responses need to be investigated.

Moreover, many investigations have proved that soil properties modulated soil prokaryotic diversity, composition, and ecological function (Tourna et al., 2008; Griffiths et al., 2011; Xu et al., 2020; Yang et al., 2021), which should regulate the response of microbes to the soil contamination stress (Bernier and Surette, 2013; Xian et al., 2015; Grenni et al., 2018). In comparison, ammonia oxidizers are more susceptible to changes in farming practices, such as tillage, fertilization, and planting (Zhou Z. et al., 2014; Azziz et al., 2016; Yan et al., 2018; Sun et al., 2019). It is also found that the distribution pattern of ammonia-oxidizers’ communities is different across distinct soils (Azziz et al., 2016; Xia et al., 2016; Liu et al., 2019). Thus, there are grounds to assume that the influence of antibiotics and heavy metals on ammonia-oxidizers’ community and function would be greatly dependent on individual soil types. Sulfonamides and copper, two common food additives largely applied in livestock farming, are frequently detected co-existence in agricultural soils as the wide application of manures (Grenni et al., 2018; Cycoń et al., 2019; Quaik et al., 2020; Yang et al., 2020), but few studies compared their toxic influence on ammonia-oxidizing microbial community and function across various soils.

To bridge the aforementioned knowledge gap, two typical groups of ammonia-oxidizing microorganisms, namely ammonia-oxidizing archaea (AOA) and ammonia-oxidizing bacteria (AOB), and the involved soil potential nitrification were chosen as biomarkers to elucidate the toxicity difference of SDZ and Cu across distinctive soils. The present study would offer an implication for the integrative management of soil contamination.

## 2. Materials and methods

### 2.1. Soil samples and materials

Black soils, Brown soils, and Desert soils, three typical soil types of China, were sampled from three farmland zones along distinct climatic gradients (Table 1). Surface soil samples (0–20 cm) were collected from a fallow plot without plants to minizine the influence of crop growing. Three sampling plots were collected from each farmland zone. Five random sites were sampled from each plot, and about 2 kg of soil was homogenized as one soil sample after picking out large stones, plant litter, and animal debris. The collected soil samples were screened (~2 mm) and then divided into two subsamples: One subsample was stored at ~4°C for microbial analysis in the next 2 weeks, and the other one was air-dried for the analysis of soil properties. Soil type and the selected soil physicochemical properties are listed in Table 1.

**Table 1:** Soil type and its selected physicochemical properties (*n* = 3).

Sites	Jilin	Shandong	Xinjiang
Location	124.40°E, 43.10°N	120.17°E, 36.80°N	87.40°E, 43.97°N
Climatic zones	Humid area	Semi humid area	Arid area
Crop	Corn	Wheat/Peanut	Peanuts
Soil type	Black soil	Brown soil	Desert soil
pH	5.95 ± 0.18a	5.75 ± 0.09a	7.77 ± 0.25b
Clay (%)	7.08 ± 0.51b	0.51 ± 0.08a	18.22 ± 3.01c
SOC (g/kg)	26.32 ± 2.97b	16.50 ± 0.77a	18.60 ± 1.99a
CEC (cmol/kg)	33.34 ± 2.92c	21.00 ± 2.90b	13.65 ± 1.79a
TN(g/kg)	1.65 ± 0.23b	2.02 ± 0.35c	1.37 ± 0.16a
NO_3_^−^-N(mg/kg)	3.37 ± 0.60b	1.39 ± 0.09a	3.83 ± 0.21c
NH_4_^+^-N(mg/kg)	10.12 ± 1.03c	7.24 ± 0.05b	5.86 ± 0.36a
Cu (mg/kg)	15.44 ± 1.87a	26.32 ± 3.0b	21.63 ± 1.65b
SDZ (mg/kg)	0.58 ± 0.07	--	--

--, SDZ was not detected; lowercase letters indicate the significance between different soils.

Sulfadiazine (AR, ≥ 98.6%) was purchased from Shanghai Yuanye Biological Technology Co. Ltd. (Shanghai, China) and prepared in a stock solution (40 mmol/l) with ultra-pure water after being dissolved by methanol (HPLC grade). A total of 1,000 mg/l of Cu^2+^ stock solution was prepared with CuSO_4_ in ultra-pure water. The reagents, except methanol, are all of the analytical grade and purchased from Sinopharm Chemical Reagent Co., Ltd. (Shanghai, China).

### 2.2. Soil property analysis

Multiple soil abiotic variables were determined according to the protocols described in the reference (Lu, 1999; Carter and Gregorich, 2007): soil pH was measured using a suspension of 1:2 soil/water with a glass electrode (FE22, Mettler Toledo, Shanghai, China); soil organic carbon (SOC) was determined by acid dichromate wet oxidation method; total N was determined by the Kjeldahl method; soil inorganic nitrogen (NH_4_-N and NO_3_-N) was extracted with 1 M KCl and analyzed by a flow injection analyzer (SAN++, Skalar, Netherlands). The clays were analyzed by a laser particle size analyzer (Rise 2008, Rise, China) after being scattered with sodium hexametaphosphate. The cation exchange capacity (CEC) was measured by analyzing the concentration of exchangeable cations in ammonium acetate extract of soils with an inductively coupled plasma-atomic emission spectrometry (ICP-AES, Optima 5300DV, Perkin Elmer). Cu in soils was determined with an inductively coupled plasma atomic emission spectrometer (ICP-MS, Vista MPX, United States, Varian) after digested by HCl, HNO_3_, and HF (8 ml, 3:9:4 v1/v2/v3) in a microwave digestion system (ETHOS 1, Italy, Milestone; Hu et al., 2011; Liao et al., 2019a,b). For the analysis of SDZ in soil, the method of Zhou A. et al. (2014) was employed with a modification, and the detailed procedure was described in the previous report (Liao et al., 2019a,b).

### 2.3. Incubation experiment

A soil sample of 50 g was added to a plastic bottle (250 ml) and then pre-incubated in dark at 25 ± 2°C for soil microbial recovery after adjusting water content to 50% of the field water-holding capacity [WHC, determined by Keen’s box method (Kumar et al., 2018)]. At the end of 7 days’ pre-incubation, the soils were spiked with SDZ and CuSO_4_, respectively, to their designed concentrations. Then, all soils were thoroughly mixed, and each soil treatment was split into two subsamples: a subsample amended with 100 mg NH_4_-N/kg fresh soil using a stock solution of (NH_4_)_2_SO_4_ and additional deionized water to reach soil moisture of 60% of WHC for soil potential nitrification rates (PNRs) analysis according to the ISO method (Rusk et al., 2004) with slight modification, seen in our prior publications (Liu et al., 2014; Liao et al., 2019a,b); another one, used for the *amoA* gene analysis, was humidified directly with deionized water to 60% of WHC and incubated for another 14 days to keep the same incubating days with soil PNR. During the whole incubation, soil moisture was maintained at 60% of WHC by adding regularly sterilized ultra-pure water.

In this study, 5 μmol/kg of SDZ was chosen as the added rate according to the previous reports (An et al., 2015; Qiao et al., 2018), but a 200 mg/kg added rate of Cu was used based on risk control standards for soil contamination of agricultural land (GB15618-2018, China). Both single and combined treatments of SDZ and Cu were performed for each soil, which was designed as S5 for the single spiking of 5 μmol/kg SDZ, C2 for the single spiking of 200 mg/kg Cu^2+^, and C2S5 for the combined spiking of 5 μmol/kg SDZ and 200 mg/kg Cu^2+^. The soil treatments without any addition of Cu or SDZ were used as the control treatment designed as CK for each soil. Each soil treatment was conducted in triplicates.

### 2.4. Soil DNA extraction and *amoA* genes analysis

The fresh soil samples were transported in iced boxes to Lc-Bio Technology Co., Ltd. (Hangzhou, China) for DNA extraction, PCR amplification, sequencing, and taxonomic assignment (Liao et al., 2019a,b).

In brief, the total DNA was extracted from 0.25 g of each soil sample with the PowerSoil™ DNA isolation kit (TianGen Biotech, Beijing, China). The fluorescence quantitative PCR (qPCR, SYBR Green I) was conducted in triplicate on a real-time ABI7500 (Applied Biosystems, Warrington, United Kingdom) using the primer amoA-1F/amoA-2R for ammonia-oxidizing bacteria (AOB) and CrenamoA23F/A616R for ammonia-oxidizing archaea (AOA) (Supplementary Table S1). The standard curves of both AOA-*amoA* and AOB-*amoA* were drawn as described previously (Liao et al., 2019a,b). Quantification of *amoA* genes of AOA and AOB was conducted by comparing the values of cycle threshold (Ct) with the standard curves, respectively.

The primers used for PCR amplification were the same as the qPCR as well as the amplification systems and conditions. After purification using the SanPrep® quick PCR purification kit, pyrosequencing working of the target PCR products was accomplished on the Illumina Miseq PE300 sequencing platform (Illumina, Inc., CA, United States), and each treatment was performed in triplicate. The raw sequences were quality-filtered and processed using QIIME v2.0. Obtained sequences from different soil samples were, respectively, clustered into the operational taxonomic unit (OTU) at 97% sequence similarity using UPARSE (Version 7.1) after chimera detection using USTARCH7.0. The archaeal and bacterial OTUs were classified by using the ribosomal database project (RDP) classifier against the functional genomics resource (FGR) functional gene database.

### 2.5. Data analysis

The software package SPSS 18.0 (SPSS, Inc., United States) was used for data statistical analysis. The significant differences between soil treatments were identified by the one-way variance analysis (ANOVA) with Tukey’s HSD *post hoc* test at a *value of p* of <0.05 significant level. The principal component analysis (PCA) and the canonical correlation analysis (CCA) were used to account for the influence of soil properties on the variation of soil PNR and ammonia-oxidizers’ community in soils after being treated with SDZ and Cu.

## 3. Results

### 3.1. Variation of soil PNR and Amo genes’ abundance in three soils under the stress of SDZ and Cu

**Figure 1:**
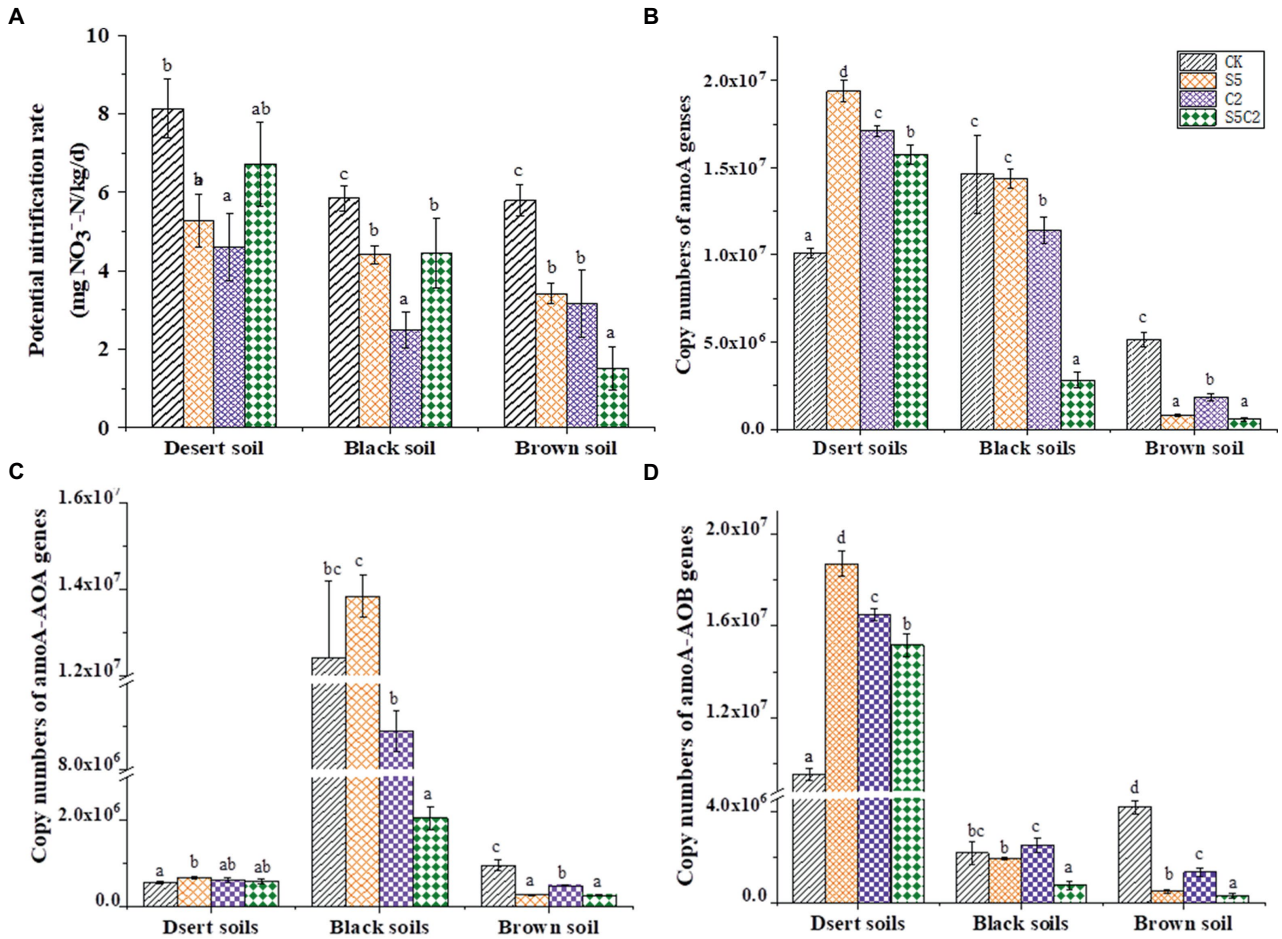
Variation of soil PNR **(A)**, the abundance of the total *amoA* genes **(B)**, AOA groups **(C)**, and AOB groups **(D)** in different soil treatments (Error bar is standard deviation; CK, control treatment; S5, 5 μmol/kg SDZ treatment; C2, 200 mg/kg Cu^2+^ treatment, and C2S5, 5 μmol/kg SDZ + 200 mg/kg Cu^2+^ treatment).

As shown in Figure 1, soil PNRs were all significantly (df = 3, *F* = 1031.84, *p* ≤ 0.05) inhibited in soils treated with SDZ and Cu singly or simultaneously; moreover, this inhibition was different across three tested soils. In detail, for the single exposure of SDZ, the highest decrease in soil PNR was observed in Brown soils, while the highest decrease of soil PNR was observed in Black soils when exposed to Cu. The combination of SDZ and Cu showed an antagonistic inhibition on soil PNR in Desert and Black soils, but there was a synergetic inhibition in Brown soils.

In view of *amo*A gene abundance (Figure 1B), it was a significant proliferation (df = 3, *F* = 10.45, *p* ≤ 0.05) in Desert soils, while a significant inhibition (df = 3, *F* = 12.36, *p* ≤ 0.05) in Brown soils was observed when exposed to SDZ and Cu singly or simultaneously. In Black soils, *amo*A gene abundance was little changed with the single application of SDZ, while it was a synergetic inhibition to the combined exposure of SDZ and Cu. Similar results were also found for the individual AOA or AOB groups (Figures 1C,D). These results indicated that the abundance of ammonia-oxidizing microorganisms might not always agree with the variation of soil PNR in distinct soils under the single or combined stress of SDZ and Cu.

### 3.2. Shift of ammoxidation community in three soils to the exposure of SDZ and Cu

**Figure 2:**
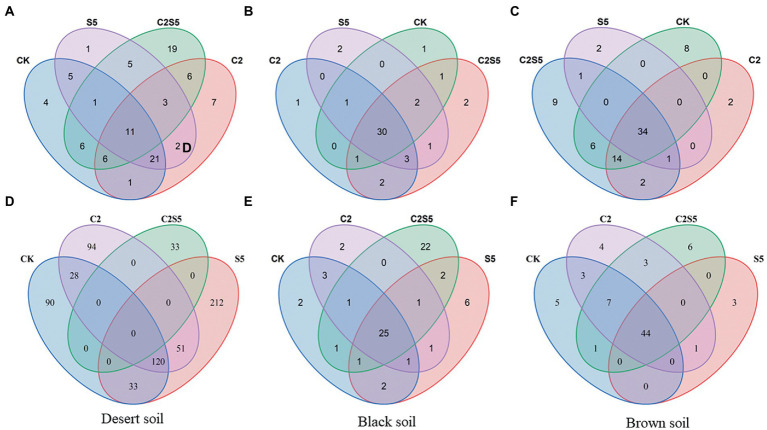
Venn diagram of overlapping AOA **(A–C)** and AOB **(D–F)** communities between different treatments of each investigated soil (CK, control treatment; S5, 5 μmol/kg SDZ treatment; C2, 200 mg/kg Cu^2+^ treatment, and C2S5, 5 μmol/kg SDZ + 200 mg/kg Cu^2+^ treatment).

The values of Chao and Shannon indexes of the AOA and the AOB groups were originally different among the three tested soils (Table 2). Moreover, the single or combined application of SDZ and Cu did not cause an obvious change in the richness and diversity of the AOA and the AOB groups in most of the cases, as based on Chao and Shannon indexes that had no significant variation in most of the soil treatments, except in Brown soils treated with SDZ alone (Table 2). However, various alterations in communities’ composition of the AOA and AOB groups were found across three tested soils to the same exposure of SDZ and Cu singly or simultaneously (Figure 2). In detail, among the different treatments of SDZ and Cu, there were only 7% of the AOA communities in common, while it was zero for the AOB community in Desert soils, but there were remained at least 25% in common for the AOA and AOB communities in Brown and Black soils (Figure 2).

**Table 2:** Communities’ diversity of ammonia oxidizers in each soil treatment.

Soils	Treatment	*amoA*-AOA			*amoA*-AOB		
		Coverage	Shannon	Chao	Coverage	Shannon	Chao
Desert soils	CK	0.999	3.15 ± 0.11a	69.44 ± 10.5a	0.997	3.94 ± 0.21b	393.5 ± 68.5a
	S5	0.999	3.05 ± 0.22a	61.05 ± 2.62a	0.996	3.91 ± 0.28b	451.8 ± 97.8a
	C2	0.999	3.33 ± 0.07a	73.71 ± 5.76a	0.997	3.51 ± 0.32b	327.7 ± 106a
	C2S5	0.999	2.86 ± 0.28a	60.6 ± 9.65a	0.999	3.39 ± 0.30a	387.3 ± 50.8a
Black soils	CK	0.9999	1.78 ± 0.13b	35.03 ± 4.24a	0.9999	1.4 ± 0.07a	39.42 ± 2.71a
	S5	0.9998	1.83 ± 0.07b	39.12 ± 9.90a	0.9999	2.07 ± 0.07b	37.17 ± 6.84a
	C2	0.9997	1.92 ± 0.07b	46.38 ± 6.54a	0.9999	1.55 ± 0.07a	35.5 ± 2.12a
	C2S5	0.9999	1.52 ± 0.19a	38.04 ± 10.90a	0.9999	2.02 ± 0.03b	43.6 ± 13.58a
Brown soils	CK	0.9998	1.25 ± 0.37a	59.25 ± 6.72b	0.9999	2.07 ± 0.04a	61.71 ± 1.82b
	S5	0.9999	1.99 ± 0.24a	36.5 ± 6.36a	0.9999	2.42 ± 0.04b	44.6 ± 3.39a
	C2	0.9998	1.03 ± 0.36a	49.53 ± 7.54ab	0.9999	2.02 ± 0.13a	60.75 ± 3.89bc
	C2S5	0.9999	1.59 ± 0.32a	66.86 ± 1.79bc	0.9999	2.45 ± 0.05b	66.01 ± 2.83bc

CK, control treatment; S5, 5 μmol/kg SDZ treatment; C2, 200 mg/kg Cu^2+^ treatment, and C2S5, 5 μmol/kg SDZ + 200 mg/kg Cu^2+^ treatment. The lowercase letters a, b, c, and d indicate the significance between different treatments of each soil type.

**Figure 3:**
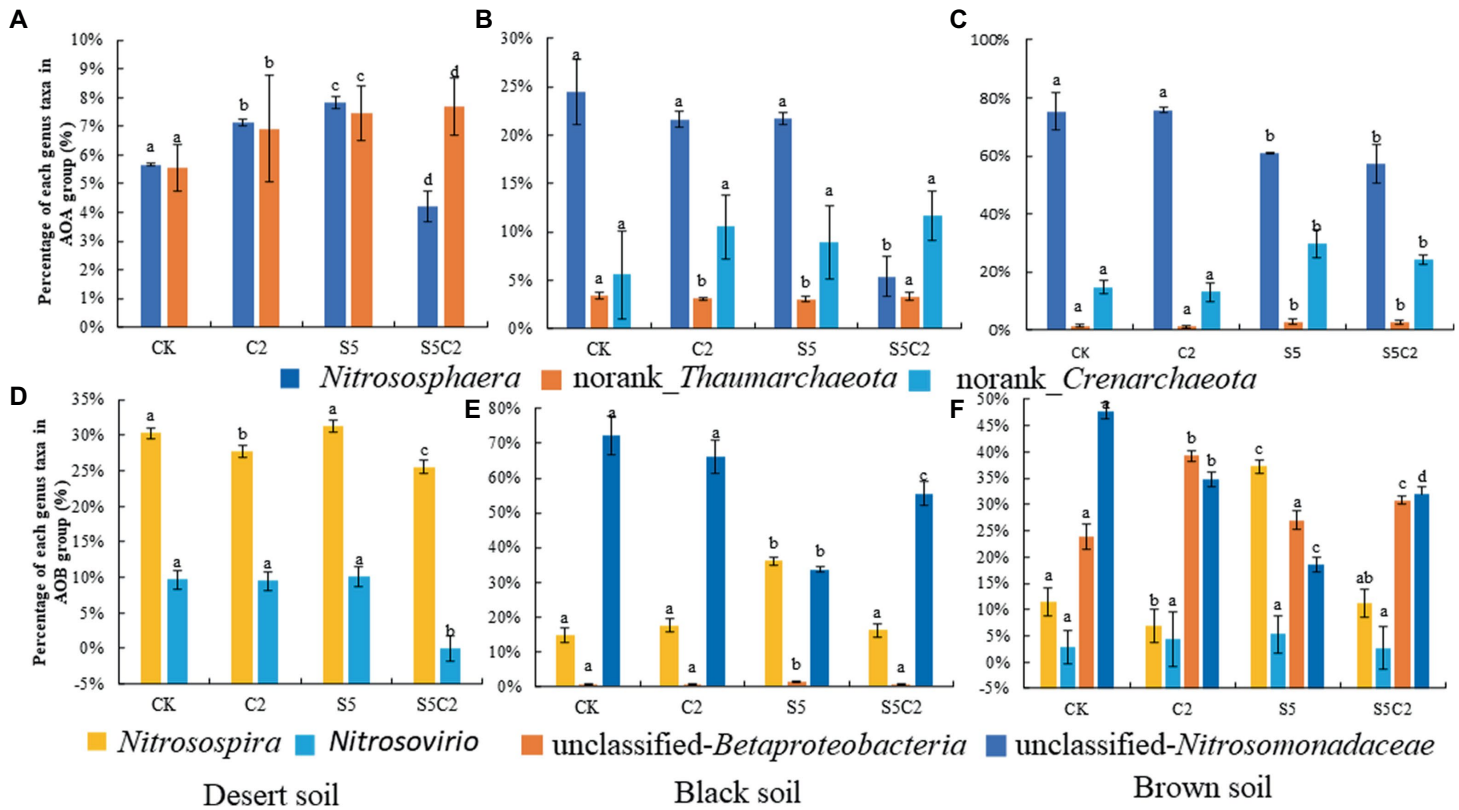
Relative abundance of the dominant AOA genera **(A–C)** and AOB **(D–F)** genera in different soil treatments (Error bar is standard deviation; CK, control treatment; S5, 5 μmol/kg SDZ treatment; C2, 200 mg/kg Cu^2+^ treatment, and C2S5, 5 μmol/kg SDZ + 200 mg/kg Cu^2+^ treatment).

Moreover, in view of the individual genus, various responses were also found among three tested soils to the exposure of SDZ and Cu singly or simultaneously, that is, the genus *Nitrososphaera* of the AOA group was significantly (df = 3, *F* = 12.90, *p* ≤ 0.05) proliferated in Desert soil, but it was inhibited in Black and Brown soils with the single application of SDZ or Cu, although the synergistic inhibition was all found in these three soils to the combined exposure of SDZ and Cu. In addition, both norank_*Thaumarchaeota* and norank_*Crenarchaeota* of the AOA group were mostly bolstered in three tested soils under the single or combined stress of SDZ and Cu (Figure 3A).

In contrast to the AOA group, the individual genus response of the AOB group was more variable across three tested soils to the single or combined exposure of SDZ and Cu (Figure 3B), that is, *Nitrosospira* was bolstered up in three tested soils when exposed to SDZ, but it was significantly inhibited in Desert and Brown soils while increased in Black soils, to the single exposure of Cu. However, unclassified *Betaproteobacterias* and *Nitrosomonadanceae* were all significantly inhibited in Black and Brown soils to the single exposure of SDZ or Cu. However, for the combined exposure of SDZ and Cu, the synergistic effect was observed on a specific genus of AOB in Desert soils, while there was an antagonistic effect in Black and Brown soils.

### 3.3. The PCA results of different treatments for three tested soils

**Figure 4:**
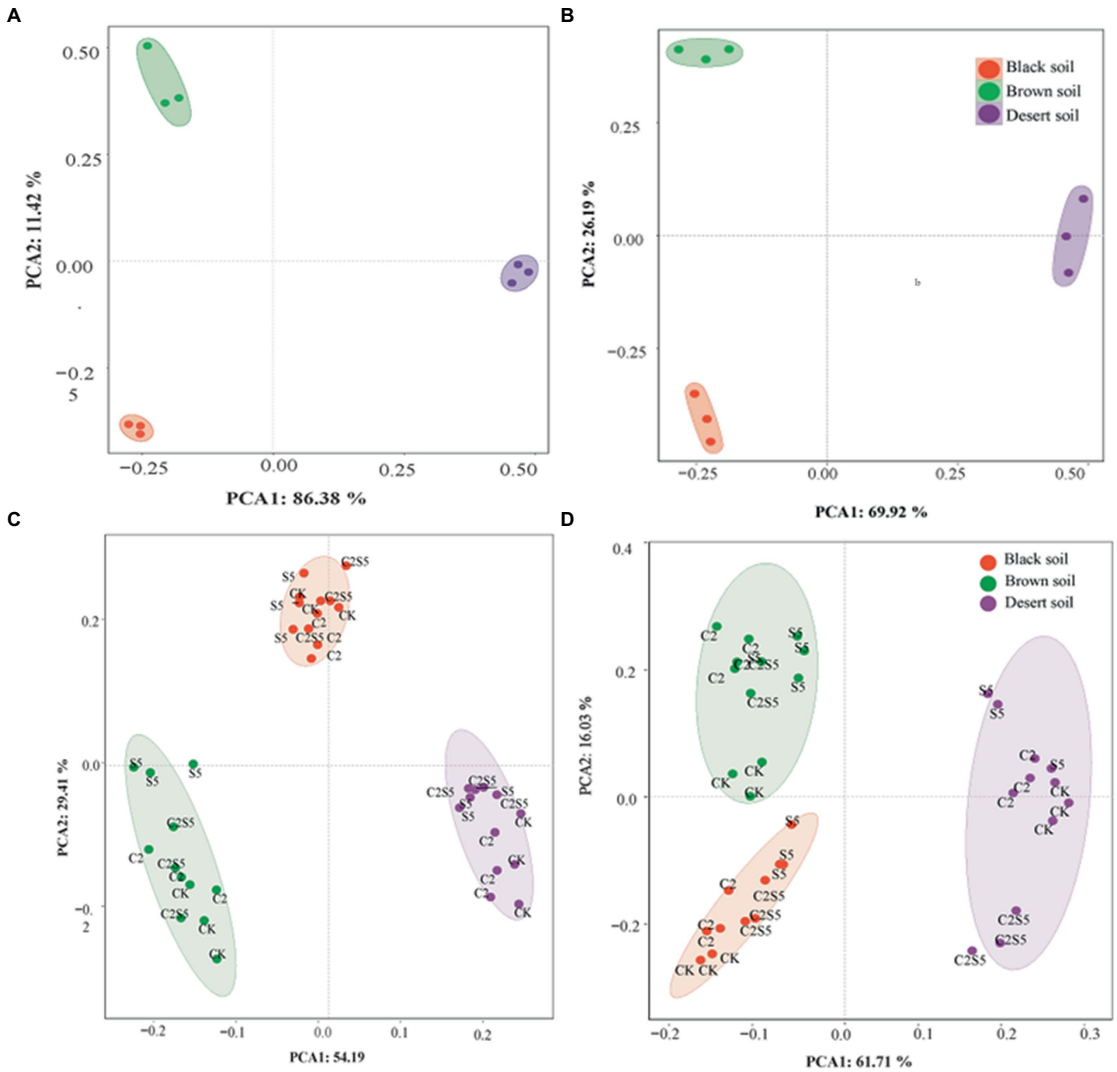
Grouping soils according to **(A)** basic soil properties from Table 1; **(B)** PNR and community abundance, diversity, and individual taxa of ammonia-oxidizing microorganism in control soil treatments from Figures 1–3 and Table 2; **(C)** PNR and community abundance, diversity and individual genus taxa of AOA in treated soils with SDZ and/Cu from Figures 1–3 and Table 2; **(D)** PNR and community abundance, diversity, and individual genus taxa of AOB in treated soils with SDZ and/Cu from Figures 1–3 and Table 2 (CK, control treatment; S5, 5 μmol/kg SDZ treatment; C2, 200 mg/kg Cu^2+^ treatment, and C2S5, 5 μmol/kg SDZ + 200 mg/kg Cu^2+^ treatment).

Principal components analysis (Figure 4) indicated that the separation of soil types by the first two PC derived from all measured biomarkers of the ammonia-oxidizing process (Figure 4B) corresponds to the distribution derived from basic soil properties (Figure 4A). Moreover, the similar soil distribution (Figures 4C,D) was also documented according to the individual AOA and AOB group in soils exposed to SDZ and Cu singly or simultaneously, which corresponds their variable variation across different soil treatments (Figures 1–3; Table 2). These results suggest that soil properties control the toxic effect of SDZ and Cu on soil ammonia-oxidizing microbial communities and function.

## 4. Discussion

### 4.1. Variable impact of SDZ on ammonia-oxidizing microorganisms across different soils

**Figure 5:**
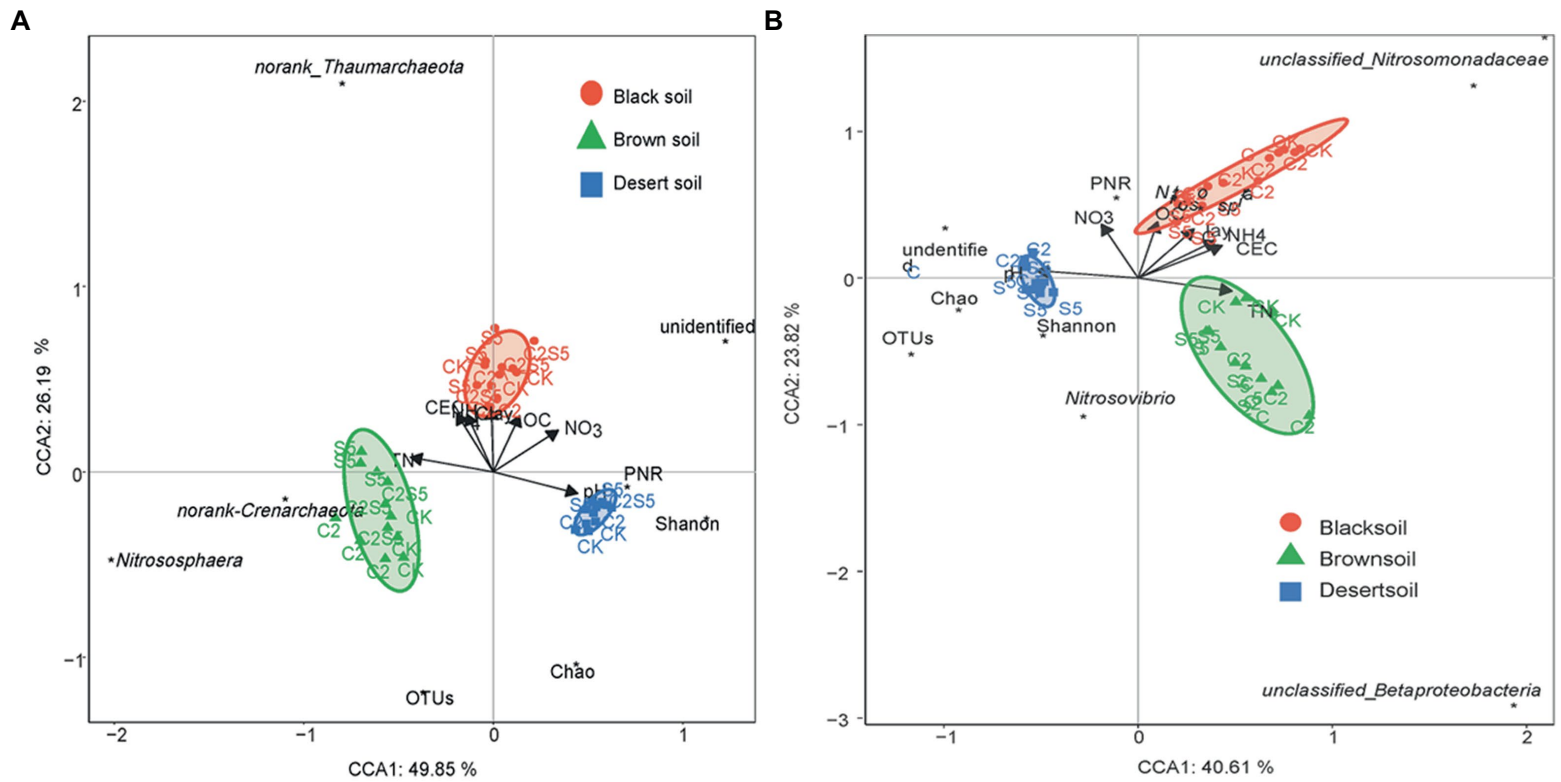
Canonical plots of discriminant analysis on soil parameters and all measured biomarkers of the ammonia-oxidizing process (**A**, for AOA groups; **B**, for AOB groups; CK, control treatment; S5, 5 μmol/kg SDZ treatment; C2, 200 mg/kg Cu^2+^ treatment, and C2S5, 5 μmol/kg SDZ + 200 mg/kg Cu^2+^ treatment).

As the critical role in the turnover of soil nitrogen, ammonia-oxidizing archaea and bacteria as well as the related soil nitrification are widely chosen to evaluate the ecological risk of soil contamination (Xu et al., 2016; Liao et al., 2019a,b; Wang L. et al., 2019). Moreover, in addition to the exposure rate and time, soil properties are proved to be non-negligible in modulating the response of soil PNR to soil contaminants’ disturbance (Kotzerke et al., 2008; Toth et al., 2011; Ma et al., 2016; Wei et al., 2018). In the present study, the variable decrease of soil PNR was also found across different soils to the single exposure of SDZ, although there was a consistent inhibition in all the soil SDZ treatments (Figure 1A). The further CCA also exhibited a significantly dependent relationship between soil properties and PNR (Figure 5). No similar correspondence was found for the response of *amo*A gene abundance across three tested soils with the application of SDZ, even counting on the individual AOA or AOB group (Figures 1B–D). It proves that the single response of PNR could not be translated into the consistent effects of SDZ on the ammonia-oxidizing microbial community in soils. This result is different from the previous reports that SDZ had a similar influence on soil nitrification function and the involved ammonia-oxidizing microbial community (Ding and He, 2010; Xing and Jin, 2018). Moreover, along with a diverse variety of *amo*A gene abundance (Figure 1), variable responses in the community diversity, composition, as well as the specific taxa of AOA and AOB group were also found across three tested soils when exposed to SDZ (Figures 2 3; Table 2). These inconsistent findings might be related to soil properties controlling microbial composition and activity (Bernier and Surette, 2013; Grenni et al., 2018; Yang et al., 2020). Similar findings were also previously reported that microbial responses to pharmaceuticals in agricultural soils depended on soil types (Frková et al., 2020), as soil properties regulating their sorption and dissipation in soils, in addition to controlling microbial composition in soils (Kodešová et al., 2020). Moreover, the present study also found that the same separation of soil types by two first PC derived from all biomarkers of ammonia-oxidizing microorganisms corresponded to the distribution derived from basic soil properties (Figure 4), and the significant dependent relationship between soil properties and ammonia-oxidizing microbial community and function (Figure 5). Contrary to the stimulatory effects on the abundance and diversity of AOA and AOB groups, the single application of SDZ in three tested soils caused a well-consistent inhibition on soil PNR and the specific genus taxa of AOA or AOB (Figures 1A, 3). It might suggest that the specific genus taxa of ammonia-oxidizing microorganisms modulate the response of soil PNR to the exposure of antibiotics, in addition to soil properties (Toth et al., 2011; Ma et al., 2016; Wei et al., 2018).

### 4.2. Combined effect of SDZ and Cu on ammonia-oxidizing microorganisms across soils

Compared to the single exposure to the antibiotic, their combination with heavy metals could cause a higher inhibition of the abundance and function of ammonia-oxidizing microorganisms (Xu et al., 2016; Wang X. et al., 2019). In the present study, to the combined exposure of SDZ and Cu, a consistent inhibition on PNR was found in three tested soils, but a variable interaction was also found across three tested soils (Figure 1A). It was previously found that the functional redundancy of soil PNR also could overspread the toxicity of contaminants on ammonia-oxidizing community (Ruyters et al., 2013). To the combined exposure of SDZ and Cu in three tested soils, no consistent correspondence was also found considering the consistent inhibition of soil PNR (Figure 1A) versus the diverse variation of *amo*A genes abundance (Figures 1B–D) in the present study. In addition, no consistent correspondence was also found in community abundance, diversity, and the specific genus taxa of soil ammonia-oxidizing microorganisms across three tested soils to the combined exposure of SDZ and Cu (Figures 1 3; Table 2). The variable changes in the microbial community were previously investigated across different soils to antibiotic and heavy metal contamination (Grenni et al., 2018; Wang L. et al., 2019), and it was thought to be associated with the biogeographic distribution of soil ammonia-oxidizing microorganisms (Liu et al., 2019; Clark et al., 2021). However, the change observed in individual genus taxa of the AOA and AOB groups (Figure 3) was well corresponded to soil PNR (Figure 1A) across soils exposed to the combination of SDZ and Cu. It indicated that the combined effect of SDZ and Cu on soil nitrification might be dependent on specific genus taxa of ammonia-oxidizing microorganisms (Liu et al., 2015; Wei et al., 2018). In another word, it might be speculated that the functional redundancy of soil PNR and the corresponding specific taxa of AOA or AOB modulate the combined toxic effect of SDZ and Cu on the biological turnover of soil nitrogen (Li et al., 2021). The aforementioned diverse results also indicate that a multi-microbial targets analysis, including function, microbial communities, and the specific phylotype, is necessary to better understand the risk of joint pollution to the soil environment (Teng and Zhou, 2018). In addition, the division of soil treatments based on all the biological values under the combined stress of SDZ and Cu also closely corresponded to distribution derived from soil properties (Figure 4), especially dependent on soil pH and TN (Figure 5). It also indicates that the differentiations in the combined impact of SDZ and Cu on ammonia-oxidizing process are greatly attributed to variation in soil properties (Bernier and Surette, 2013; Xian et al., 2015; Grenni et al., 2018).

## 5. Conclusion

In summary, the single application of SDZ caused a consistent decrease in the soil nitrification rate of three tested soils, but it was more serious only in Brown soils when combined with Cu. In contrast, the single or combined impact of SDZ and Cu was variable in *amo*A gene abundance and community diversity of ammonia-oxidizing microorganisms across different soils. However, similar correspondences occurred between the PNR and the specific genus taxa of ammonia-oxidizing microorganisms among different soils exposed to SDZ and Cu singly or simultaneously. Finally, the specific phylotype of AOA or AOB plays an important role in modulating the response of soil nitrification to the contamination stress of SDZ and Cu. Thus, it should propose an integrative assessment of soil properties and multiple microbial targets in soil contamination management.

## Data availability statement

The data that support the findings of this study are available from the corresponding author upon reasonable request. All the DNA sequence data in this manuscript are deposited in the GenBank databases, accession number KFXR00000000.

## Author contributions

GH: data curation and original draft preparation. ZGW: English improvement and modification. JL, GW and FR: data curation and experiment investigation. YX, KL, and ML: visualization and editing. AL: conceptualization and methodology. HL and FW: reviewing and validation. All authors contributed to the article and approved the submitted version.

## Funding

The study was supported by the National Natural Science Foundation of China (NSFC) under Grant Nos. 42077129, 41877122, 41671322, and 42177403 and the Shandong Natural Science Foundation under Grant Nos. ZR2020ZD19 and ZR2022YQ34.

## Conflict of interest

The authors declare that the research was conducted in the absence of any commercial or financial relationships that could be construed as a potential conflict of interest.

## Publisher’s note

All claims expressed in this article are solely those of the authors and do not necessarily represent those of their affiliated organizations, or those of the publisher, the editors and the reviewers. Any product that may be evaluated in this article, or claim that may be made by its manufacturer, is not guaranteed or endorsed by the publisher.
